# Israeli pediatricians’ confidence level in diagnosing and treating children with skin disorders: a cross-sectional questionnaire pilot study

**DOI:** 10.3389/fmed.2023.1250271

**Published:** 2023-09-20

**Authors:** Nicolas Andre, Liezl Muallem, Lior Yahav, Inbal Golan-Tripto, Atar Ben Shmuel, Amir Horev

**Affiliations:** ^1^Faculty of Health Sciences, Ben-Gurion University of the Negev, Beer Sheva, Israel; ^2^Pediatrics Department, Soroka University Medical Center, Beer Sheva, Israel; ^3^Pediatric Pulmonary Unit, Soroka University Medical Center, Beer Sheva, Israel; ^4^School of Health Profession Education, Maastricht University, Maastricht, Netherlands; ^5^Pediatric Dermatology Service, Soroka University Medical Center, Beer Sheva, Israel

**Keywords:** dermatology, medical education, pediatrics, residency, self-efficacy

## Abstract

**Background:**

Pediatricians daily see large numbers of patients with skin disorders. However, they encounter limited guidance as a result of a marked deficiency in pediatric dermatologists. Hence, reevaluation of training opportunities during pediatric residency has become essential. Our aim was to evaluate the confidence level of pediatric residents and specialists in diagnosing and treating skin disorders in children and to determine career and training-related characteristics that influence it.

**Methods:**

Conducted as a cross-sectional study, we administered a questionnaire to 171 pediatricians across Israel. We assessed respondents’ self-efficacy about their ability to diagnose and treat skin disorders and collected data regarding their previous dermatology training and preferred training methods.

**Results:**

77.8% of respondents reported below or average self-efficacy scores in diagnosing and managing children with skin disorders. Older age (>40 years old; OR = 5.51, *p* = 0.019), treating a higher number of patients with skin disorders (OR = 2.96, *p* = 0.032), and having any training in dermatology, either during medical school or residency (OR = 7.16, *p* = 0.031, OR = 11.14, *p* = 0.003 respectively), were all significant parameters involved in pediatricians reporting high self-efficacy in skin disorder management.

**Conclusion:**

Most pediatric residents and pediatricians have average or below-average confidence in managing pediatric skin disorders. We suggest incorporating dermatology rotations during pediatric residency to improve young pediatricians’ self-efficacy in managing skin disorders and ultimately help pediatricians provide better care for patients presenting with dermatological conditions. These findings can ultimately help refine a pilot program in dermatology that might be implemented during pediatric residency.

## Introduction

Skin disorder complaints are a common reason for children’s visits to pediatric clinics and emergency rooms. According to current data, primary skin problem represents 4–6% of emergency room visits and up to 24% of the primary and secondary reasons for clinic visits (1–3). Frequent skin disorders in pediatric settings involve infectious skin diseases (27.6%), atopic dermatitis (AD; 17.9%), acne (14.5%), papulosquamous diseases (6.9%), and hair diseases (4.1%) (4). As a result, pediatricians are at the forefront of the diagnosis and treatment of these children, even though studies are reporting difficulties in the management of skin disorders for those physicians (5). As such, the literature reports that more than half of pediatricians in the United States refer even mild cases of AD to dermatologists (6). Common skin diagnoses such as warts, cysts, and acne were found to be diagnosed at least 10 times less frequently by primary care physicians than by dermatologists, which might ultimately affect the outcome of care (7). Finally, pediatricians tend to be more conservative when employing certain treatments, like topical corticosteroids. This circumspection is especially evident in aspects such as the potency of the treatment and its duration (7, 8), distinguishing their approach from that of dermatologists who have undergone further training through a pediatric dermatology fellowship. The variations in how skin disorders are managed, diagnosed, and treated result in elevated rates of referrals (6, 9, 10) and contribute to a heavier workload for dermatologists. This, in turn, prolongs waiting times and exacerbates an already escalating scarcity.

The large percentage of misdiagnosed common cutaneous diseases may be due to pediatricians’ lack of confidence in dealing with the diagnosis and treatment of skin diseases in children (11, 12). A survey among graduate physicians who trained in pediatrics reported that 38% of them desired additional training in dermatology (13). This may be explained by the fact that in some countries, like Israel, dermatology rotations are not obligatory components of pediatric residency, but rather are provided as elective choices. To be more specific, pediatric residents are allotted two consecutive months of elective rotations, with each rotation lasting a minimum of 1 month. Apart from dermatology, these electives encompass other important medical fields, including otolaryngology (ear and throat), gastroenterology, cardiology, nephrology, orthopedics, and many others, to highlight just a few examples. This might result in many pediatricians finishing their residency without official guidance in diagnosing and treating pediatric skin disorders.

Self-efficacy surveys are an accepted tool for the assessment of medical personnel confidence as it tests one’s belief in their ability to achieve a particular task (7, 8, 14). Correlations between self-efficacy and clinical performance along with correlations between self-efficacy and success in different tasking, have been widely reported in the literature and provide determined support for the positive effect of self-efficacy on performance (15–17).

Considering this deficit in pediatricians’ training, we aimed to evaluate the confidence level of residents and pediatricians in diagnosing and treating skin disorders in children, based on a self-efficacy survey among pediatric residents and specialists. Moreover, we aimed to characterize the high self-efficacy group, according to their demographic data, medical training and dermatology training.

We hypothesize that identifying career and training-related attributes influencing the self-confidence of pediatricians in diagnosing and treating skin disorders in children could facilitate their inclusion in residency programs and enhance the educational experience for pediatricians.

## Methods

Conducted as a cross-sectional pilot study, we administered a questionnaire to Israeli pediatricians between April 2022 and June 2022. The Institutional Review Board of Soroka University Medical Center reviewed our study and granted an exemption, as no medical data regarding patients was included and that responses were anonymous.

### Participants

Our target population were all pediatricians, whether residents or specialists, who either worked in hospitals or community clinics in Israel. Physicians with no pediatric specialization but involved in treating children, such as pediatric dermatologists or general practitioners, along with residents of other specialties than pediatrics, were all excluded. Preliminary consent from all participants was obtained prior to completing the questionnaire. Our data was collected via a Google-based questionnaire that was sent to the participants by e-mail or text message. No incentives of any kind were given to respondents. All the data obtained were anonymous and participants’ confidentiality was maintained throughout the study.

### Questionnaires

Our questionnaire (Appendix) aimed to identify characteristics involved in the high or low self-efficacy of pediatric residents and specialists when encountering skin disorders.

Three sections composed our questionnaire. The first section dealt with general data regarding demographics. This included gender, age, marital status, religion, location of medical school (Israeli/non-Israeli medical school), medical status (resident/specialist/fellow/pediatrician with sub-specialty), stage and years of residency, current place of work (community/hospital/private clinics), and job seniority. The next section aimed to assess the respondent’s self-efficacy about their ability to diagnose and treat skin disorders. Evaluation of responses was based on a scale from 1 to 5, with one being the lowest score, five the highest score, and three the average. The last section examined the teaching methods followed by the respondent during their training and during their continuing education on skin disorder management. Our questionnaire proposed eight teaching/training methods as best suited for appropriate training. These were lectures, continuing medical education, online program, dermatology rotation, frontal convention/conference, online convention/conference/webinar, dermatology research, experts case discussions, and “other.” Additionally, participants were asked to estimate their ability to tutor colleagues on the management of skin disorders in children. This approach, aimed at gaging their self-assuredness in diagnosing and treating skin disorders, indirectly utilized the confidence linked to their teaching abilities to provide a more accurate assessment. Moreover, respondents were asked detailed questions about information they would use and management steps they would take when encountering a particular skin disorder (online search engine, medical literature, and consult/refer to a dermatologist). Finally, respondents were asked to recommend the methods they thought best to improve dermatology training in residency and be better prepared for the management of skin disorders.

### Statistical analysis

Percentages were used to describe categorical data while continuous variables were presented as mean ± SD. Statistical significance was defined as a value of *p* ≤ 0.05. The Pearson’s chi-square test was used to compare nominal variables while Student’s *t*-test or One-Way ANOVA was used to compare continuous variables that failed to respect normal distribution. Ordinal and continuous variables that did not meet parametric criteria were compared using Kruskal-Wallis or Mann–Whitney tests. Analyses were performed using the IBM SPSS software version 22.

## Results

Out of the 350 pediatricians to whom we distributed the questionnaire, 171 respondents completed it (48.86%). The average age of our study population was 41.05 ± 10.55 years. The population was composed in majority of females (59.4%). 39.1% of respondents were residents, 60.9% were board-certified specialists which included pediatric specialists (29.6%), fellows (training for sub-specialty; 9.5%), and pediatric specialists who completed a subspecialty (21.9%). A majority (78.4%) of our respondents went to medical school in Israel and 70.2% worked in a hospital setting. Details about the demographic characteristics of our population are presented in Table 1.

**Table 1:** Baseline characteristics of the study population.

Characteristics	*N* (%)
Age	41.05 ±10.55
Gender	
Males	69 (40.6%)
Females	101 (59.4%)
Religion	
Jewish	134 (78.4%)
Muslim	21 (12.3%)
Christian	2 (1.2%)
Other/Do not want to answer	14 (8.2%)
Marital status	
Married	142 (83%)
Bachelor	21 (12.3%)
Divorced	2 (1.2%)
Other/Do not want to answer	6 (3.5%)
Country of MD school	
Israeli medical school	134 (78.4%)
Non-Israeli medical school	37 (21.6%)
Medical status	
Resident	66 (39.1%)
Specialist	50 (29.6%)
Specialist with sub-specialty	37 (21.9%)
Fellowship	16 (9.5%)
Stage in residency	
Before stage 1 exam	49 (70%)
After stage 1 exam	4 (5.7%)
After stage 2 exam	17 (24.3%)
Years of residency	
1	12 (17.9%)
2	13 (19.4%)
3	25 (37.3%)
4	11 (16.4%)
5	6 (9%)
Current place of work	
Hospital	120 (70.2%)
Community based clinic	66 (38.6%)
Private practice	7 (4.1%)
Number of years as a pediatrician	10.87 ±10.84

Baseline characteristics of the study population.

Over two thirds of respondents reported below or average self-efficacy scores in diagnosing and managing children with skin disorders (77.8%), while 22.2% of them stated to be above average (Table 2).

**Table 2:** Fundamental self-assessment skills of participants.

How do you estimate your skills in diagnosing and managing a skin disorder in children?	*N* (%)
1	7 (4.1%)
2	40 (23.4%)
3	85 (50.3%)
4	37 (21.6%)
5	1 (0.6%)

Fundamental self-assessment skills of participants.

Our study population was subsequently divided into two groups according to their self-efficacy level—average or below (≤3) and above average (4, 5). This was performed to identify parameters involved in higher self-efficacy scoring. The higher self-efficacy group was above 40 years of age (26.8 vs. 15.3% under 40, *p* = 0.019), were already specialists (34 vs. 4.5% of residents, *p* < 0.001), had more years of experience in evaluating children with skin disorders (*p* = 0.002), and was trained in dermatology during their medical school or residency (*p* = 0.031 and *p* = 0.03 respectively). Gender, place of medical school, location of residency, stage in the residency, and current working place did not differ between the groups (Table 3). Additionally, participants were inquired about dermatology training methods and dermatology rotation length during residency. Most high self-efficacy participants reported to have been trained by more than three training methods (85.8 vs. 58.8%, *p* < 0.001) with longer than a week-long dermatology rotation during residency (72.7 vs. 52.8%, *p* = 0.07).

**Table 3:** Characteristics of pediatricians stratified according to self-efficacy scoring.

Characteristic	Self-efficacy scoring ≤ average	Self-efficacy scoring > average	*p* value (univariate analysis)	*p* value (multiple regression)
Age				
40 or under	94 (84.7%)	17 (15.3%)	**0.003**	**0.019**
Above 40	36 (63.2%)	21 (26.8%)		
Gender				
Male	52 (75.4%)	17 (24.6%)	0.45	
Female	81 (80.2%)	20 (19.8%)		
Country of MD school				
Israeli medical school	104 (77.6%)	30 (22.4%)	0.92	
Non-Israeli medical school	29 (78.4%)	8 (21.6%)		
Place of pediatric training				
With dermatology depart.	73 (73.7%)	26 (26.3%)	0.12	
Without dermatology depart.	54 (84.4%)	10 (15.6%)		
Medical status				
Resident	63 (95.5%)	3 (4.5%)	**<0.001**	0.550
Specialist	68 (66%)	35 (34%)		
Stage in residency				
Before stage 1 exam	46 (93.9%)	3 (6.1%)	0.51	
After stage 1 exam	4 (100%)	0		
After stage 2 exam	17 (100%)	0		
Estimated children with skin disorder in 1 year				
Last than 10	14 (100%)	0	**0.002**	**0.032**
10–20	35 (94.6%)	2 (5.4%)		
21–40	35 (74.5%)	12 (25.5%)		
More than 40	47 (67.1%)	23 (32.9%)		
Dermatology training in medical school or internship				
Yes	61 (69.3%)	27 (30.7%)	**0.005**	**0.031**
No	71 (87.7%)	10 (12.3%)		
Dermatology training in residency				
Yes	20 (52.6%)	18 (47.4%)	**<0.001**	**0.003**
No	112 (84.8%)	20 (15.2%)		
How many training methods				
Less than three methods	103 (85.8%)	17 (14.2%)	**<0.001**	0.455
Three methods or more	30 (58.8%)	21 (41.2%)		
Dermatology rotation				
Week	40 (72.7%)	15 (27.3%)	**0.07**	0.926
Longer	19 (52.8%)	17 (47.2%)		

Characteristics of pediatricians stratified according to self-efficacy scoring. The bold values represent significant results with a *p*-value under 0.05.

Multivariable logistic regressions performed on these significant parameters were conducted in order to reinforce their significance after controlling all other variables. These showed that being older (>40 years old; OR = 5.51, *p* = 0.019), treating higher number of patients with skin disorders (OR = 2.96, *p* = 0.032) and having any training in dermatology, either during medical school or residency (OR = 7.16, *p* = 0.031, OR = 11.14, *p* = 0.003, respectively), were significantly higher in the high self-efficacy group (Table 3).

Additionally, we observed that most participants reporting low self-efficacy in managing skin disorders were also reporting average/low ability to teach their colleagues (86.8 vs. 5.3%, *p* < 0.001), and vice versa (Table 4).

**Table 4:** Self-assessment skills of participants as tutors.

		Self-efficacy scoring ≤ average	Self-efficacy scoring > average	*p* value
How do you estimate your ability to teach colleagues to manage skin disorders in children?	Average or below	131 (86.8%)	20 (13.2%)	<0.001
	Above average	1 (5.3%)	18 (94.7%)	

Self-assessment skills of participants as tutors.

Finally, none of the proposed resources pediatricians would be expected to use when encountering a particular skin disorder were significantly more prevalent by pediatricians regardless of their self-efficacy scores in managing skin disorders in children.

## Discussion

In our cross-sectional study involving 171 pediatric physicians, our findings indicated that a significant portion of the respondents expressed an average or below-average level of confidence in diagnosing and managing skin disorders in children. This is noteworthy considering that a majority of the respondents were board-certified pediatric specialists and the majority were also employed within hospital settings. However, certain characteristics such as older age, experience in managing high volumes of patients with skin diseases and prior exposure to dermatology during primary training positively influenced pediatricians’ confidence in the matter. Additionally, physicians with high self-efficacy also reported to be more comfortable teaching colleagues about managing these affections.

Though skin disorders are common affections seen by pediatricians, the variety found in children is so broad that it most often exceeds most primary care physicians’ managing skills (5). As a result, many of them, residents, and specialists alike, do not judge themselves competent in diagnosing and treating these dermatological conditions. To illustrate this, self-reported adequacy of residency training in the care of children with AD, in North Carolina, among dermatologists and primary care physicians, including pediatricians, shows that most of the latter reported their residency training in this area as only “enough to get by” or “inadequate” (18). This perceived feeling from pediatricians was further validated by 78 residency program directors from all over the United States who confessed a need to address educational deficiencies in the diagnosis and management of dermatological disorders by pediatric residents (19).

The striking number of pediatricians (77.8%) that feel average or below average confident in managing skin diseases could be the result of the poor exposure to dermatology during training, as mentioned above. Because our multivariable logistic regression analysis failed to find a difference in the self-efficacy scoring between residents and specialists, this suggests that not only initial training in dermatology is insufficient but also that this deficit might be further exposed after graduation. Analogously, studies focusing on how pediatric residency translates into general pediatric practice have reported shortcomings and low confidence in the management of such medical areas as mental health, sports medicine, or dermatological disorders (19, 20). Our study seems consistent with these findings and shows how deficits in proper dermatological training during residency may carry into regular practice later. This underlines therefore the need for further development of dermatology curricula during pediatric residency.

Unsurprisingly, however, it appears that experience might compensate for the lack of training residents and junior doctors experienced. First, older age (>40 years old) which is a surrogate marker for higher experience in treating children in general, was significant in the parameters involved in increasing pediatricians’ self-efficacy. This also indicates that cumulative experience treating children with various dermatological disorders gained over years of practice improves pediatricians’ confidence in managing these conditions. This appears to us as a compelling result as the average age of graduating medical school is 28 years old (21). The time lapse between graduation and attaining confidence in treating skin disorders could potentially result in pediatricians delivering less-than-optimal care to patients for a significant duration until they acquire the necessary experience. This further reinforces our claim about the necessity of reevaluating dermatology training during early years of pediatrics residency. A practical approach would be early exposure to high numbers of patients that present with skin disorders, as our findings suggest. Though the current shortage of pediatric dermatologist might impede this, exposing residents to more skin disorder patients could be achieved by elevating the status of dermatology to that of a training requirement rather than an elective, as prior studies have suggested (19).

To further illustrate this, our results show that any dermatology exposure during medical school or residency, such as suggested in our questionnaire, significantly increases pediatrician’s confidence in managing skin affections. Early experience in dermatology seems to be an effective way for physicians to gain the necessary confidence in diagnosing and treating skin disorders. Though our preliminary results showed that high numbers of different training methods along with dermatology rotations longer than a week were effective educational ways for an increase in self-efficacy, multiple regression on these parameters did not reach significance. However, improving self-efficacy via training methods is a dynamic process that involves a multifaceted approach which may not be reflected in our analysis. Further studies exploring parameters such as the correlation between time spent in dermatology rotations and self-efficacy in skin disorder management would be necessary to better refine this aspect of dermatology education in pediatricians.

Furthermore, since research has shown a favorable link between mentorship and professional growth, increased productivity, and academic achievements (22), exploring ways to enhance confidence, such as introducing specialized training in collaborative pediatric-dermatology clinics or initiating mentorship initiatives with dermatologists supervising pediatric residents, could present valuable recommendations for both medical institutions and the enhancement of pediatric residency programs.

Our study has several strengths. First, to the best of our knowledge, our study is the first study to exhaustively analyze pediatricians’ clinical and educational characteristics involved in their confidence in treating skin disorders. Second, our study population was composed of a relatively large and diverse cohort, which included residents, specialists, fellows, and specialists with a subspecialty. This high number of respondents is a true representation of the pediatrician population which allowed for meaningful sub-analysis of the groups. Moreover, our questionnaire method may be easily replicated and can provide an accurate assessment of the study population, as all data remained anonymous.

However, our study has some limitations. First, our study population is from Israel, where a distinct national pediatric training program is in place. Consequently, this questionnaire’s findings might not apply to other countries that follow different training programs. Second, we had a limited response rate (48.86%) which might be attributed to the voluntary nature of the questionnaire, its extended length, and the significant workload experienced by both physicians in general and resident physicians in particular. Third, though it raised our compliance rate, our simplified multiple-choice questions may have prevented participants from providing complex ideas and concepts. Finally, our questionnaire has not yet been validated. Nevertheless, as a pilot study, we believe that our questionnaire can still be used in further studies that wish to assess parameters that might help improve dermatology education in pediatricians.

## Conclusion

Most pediatric residents and pediatricians have average or below average confidence in diagnosing and treating pediatric skin disorders. In the current era, pediatricians frequently encounter a significant number of patients with skin disorders. This challenge is further exacerbated by a shortage of pediatric dermatologists which results in limited guidance. Therefore, it has become imperative to reconsider training opportunities within pediatric residency programs. We suggest incorporating dermatology rotations during pediatric residency as mandatory rotations to improve young pediatricians’ self-efficacy in managing skin disorders and ultimately help pediatricians provide better care for patients presenting with dermatological conditions. These findings will ultimately help us refine a pilot program in dermatology that we plan to implement during pediatric residency. A comparable study conducted on a broader scale would be necessary to assess the confidence levels of pediatricians and even general practitioners in different regions and countries.

## Data availability statement

The raw data supporting the conclusions of this article will be made available by the authors, without undue reservation.

## Author contributions

NA: writing—original draft and writing—review and editing. LM and LY: methodology, data curation, formal analysis, and writing—review and editing. IG-T: conceptualization/design, methodology, investigation, supervision/oversight, and writing-review and editing. AB: conceptualization/design, methodology, investigation, supervision/oversight, data curation, resources, and writing-review and editing. AH: conceptualization/design, methodology, investigation, supervision/oversight, data curation, resources, funding acquisition, and writing-review and editing. All authors contributed to the article and approved the submitted version.

## Abbreviation

AD: Atopic dermatitis
