# Supplementary material for: Israeli pediatricians’ confidence level in diagnosing and treating children with skin disorders: a cross-sectional questionnaire pilot study
Source: Front Med (Lausanne). 2023 Sep 20;10:1250271. doi: 10.3389/fmed.2023.1250271 (PMC10548117; doi:10.3389/fmed.2023.1250271)
Supplement: Supplementary file 1 [file Table_1.DOCX]

**Questionnaire**

**Self-Efficacy questions:**

The following questions address the self-assessment of your skills with managing and treating skin disorders, particularly Atopic dermatitis in children.

Please rate the following 3 questions on a scale of 1-5 (1 represents much below average, and 5 represents much above average)

|  | 1. Much below average | 1. Below average | 1. Average | 1. Above average | 1. Much above average |
| --- | --- | --- | --- | --- | --- |
| How do you estimate your skills in diagnosing and managing a skin disorder in children? |  |  |  |  |  |
| How do you estimate your ability to teach colleagues to manage skin disorders in children? |  |  |  |  |  |

Please rate the following questions on a scale of 1-5 (1 represents strongly disagree to 5 represents strongly agree)

|  | 1. Strongly disagree | 1. Disagree | 1. Neutral | 1. Agree | 1. Strongly agree |
| --- | --- | --- | --- | --- | --- |
| 1. I would use online search engine to manage a case of a skin disorder |  |  |  |  |  |
| 2. I would use a medical literature information to manage a case of a skin disorder |  |  |  |  |  |
| 3. I would consult/refer to a dermatologist every case of a skin disorder |  |  |  |  |  |
| 4. I would consult/refer to a dermatologist only in a case of unclear diagnosis |  |  |  |  |  |
| 5. I would consult/refer to a dermatologist skin cases because of parents’ anxiety |  |  |  |  |  |

**Education tool:**

The following questions refer to dermatology education methods.

Please answer yes or no in the appropriate questions and choose from the multiple-choice questions according to your experience.

1. Have you had dermatology training as part of your medical school or internship? Yes/No
2. Have you had dermatology training during your residency? Yes/No
3. What is the method of training you have experienced? You may choose more than one answer
4. Lectures
5. Continuing medical education
6. Online program
7. Dermatology rotation – if yes, how long? Week/Month/2 Months/Longer
8. Frontal convention / Conference
9. Online convention / Conference / Webinar
10. Dermatology research
11. Experts case discussions
12. Other______
13. Have you felt more confident in treating skin disorders in children after experiencing those programs? Yes/no
14. Which method was most effective in improving your dermatologic abilities? You may choose more than one answer
15. Lectures
16. Continuing medical education
17. Online program
18. Dermatology rotation – if yes, how long? Week/Month/2 Months/Longer
19. Frontal convention / Conference
20. Online convention / Conference / Webinar
21. Dermatology research
22. Experts case discussions
23. Other______
24. From the methods listed below, which would you recommend adding as a mandatory curriculum in a residents training program that would positively affect their dermatology skills. You may choose more than one answer
25. Lectures
26. Continuing medical education
27. Online program
28. Dermatology rotation – if yes, how long? Week/Month/2 Months/Longer
29. Frontal convention / Conference
30. Online convention / Conference / Webinar
31. Dermatology research
32. Experts case discussions
33. Other______

**Demographic questions:**

Please complete the following questions according to your current status:

1. Age ____
2. Gender M/F
3. Religion
4. Jewish
5. Christian
6. Muslim
7. Other/Do not wish to answer
8. Marital Status
9. Bachelor
10. Married
11. Divorcee
12. Widower
13. Other/Do not wish to answer
14. Number of Children _____
15. Where did you attend medical school?
16. Israel
17. Europe
18. USA
19. Asia
20. Other
21. Place of pediatric training
22. list of different hospitals
23. Abroad
24. Medical status
25. Resident
26. Specialist
27. Fellowship
28. Specialist with sub-specialty
29. If resident, then:

- What stage in residency?

1. Before stage 1 exam
2. after stage 1 exam
3. after stage 2 exam

- What year of residency

1. 1
2. 2
3. 3
4. 4
5. 5
6. Current place of work (you can choose more than one)
7. Hospital
8. Community based clinic
9. Private practice
10. If hospital, which department: (if resident Ped. Department is automatically chosen)
11. Intensive Care Unite
12. Emergency Department
13. Neonatology
14. Day hospitalization
15. Clinics
16. Pediatric department
17. Number of years as a pediatrician (including training) ___
18. In your estimation how many children with skin disorder have you encountered in the last year?
19. Under 10
20. 10-20
21. 20-40
22. Above 40
23. In your estimation how many children with Atopic Dermatitis have you encountered in the last year?
24. Under 10
25. 10-20
26. 20-40
27. Above 40
